# Cytokine associated with severity of depressive symptoms in female nurses in Korea

**DOI:** 10.3389/fpubh.2023.1194519

**Published:** 2023-08-10

**Authors:** Yoonjoo Kim, Yanghee Pang, Hyunki Park, Oksoo Kim, Hyangkyu Lee

**Affiliations:** ^1^Department of Nursing, Yonsei University Graduate School, Seoul, Republic of Korea; ^2^Department of Nursing, College of Healthcare Sciences, Far East University, Eumseong-gun, Republic of Korea; ^3^Department of Nursing, Baekseok Culture University, Cheonan, Republic of Korea; ^4^Mo-Im Kim Nursing Research Institute, College of Nursing, Yonsei University, Seoul, Republic of Korea; ^5^College of Nursing, Ewha Womans University, Seoul, Republic of Korea; ^6^Ewha Research Institute of Nursing Science, Seoul, Republic of Korea

**Keywords:** cytokine, depression, Korea, generalized gamma regression, nurse

## Abstract

**Background:**

Depression has been associated with the risk of developing physical illnesses and diseases. Inflammatory hypotheses of immunoactive and dysregulated cytokine production have been proposed to describe this association; however, data pertaining to the high prevalence of depression among nurses are limited.

**Objective:**

This study aimed to use a comprehensive immune-profiling approach to determine whether an abnormal profile of circulating cytokines could be identified in nurses with self-reported depression and whether this profile is associated with the severity of depression.

**Methods:**

We investigated a cohort of 157 female nurses in Korea. The self-report Patient Health Questionnaire was used to measure the depression levels of nurses. In addition, peripheral blood samples were collected and used to measure the cytokine profile using the Luminex multiplexing system. Generalized gamma regression analyses were conducted to evaluate the association between cytokine and depressive symptoms.

**Results:**

Regarding severity of depressive symptoms, 28.0% of nurses had moderately severe depression while 9.6% had severe depression. Moderately-severe depressive symptoms in nurses were associated with elevated levels of interleukin-6 (*B* = 0.460, *p* = 0.003), interleukin-8 (*B* = 0.273, *p* = 0.001), and interleukin-18 (*B* = 0.236, *p* = 0.023), whereas interferon-gamma levels (*B* = −0.585, *p* = 0.003) showed the opposite profile. Participants with severe depressive symptoms presented decreased interferon-gamma levels (*B* = −1.254, *p* < 0.001).

**Conclusion:**

This study demonstrated that proinflammatory cytokines were associated with depression among nurses. This calls for early detection and intervention, considering the mechanisms linking depression to physical illness and disease.

## 1. Introduction

Depression is one of the most common and harmful mental disorders, affecting about 15–20% of the general population (1, 2). The number of depression cases reported worldwide increased by 49.9% from 1990 to 2017 (3). Generally, depression causes unhealthy behaviors, such as smoking, reduced physical activity, and excessive calorie intake (4, 5) and has been shown to be associated with increased inflammation, metabolic dysregulation, increased obesity, and worsening chronic diseases (6–8). Though several potential psychophysiological mechanisms explain this association, the inflammatory hypothesis of immune hyper-activation and dysregulated cytokine production has been widely supported (6, 9–11).

In a meta-analysis of inflammatory markers of depression, inflammatory cytokines have been shown to be representative biomarkers (12, 13). Cytokines are typically pro- and anti-inflammatory, and their balance determines the outcome of the inflammatory response (14). In a systematic review and meta-analysis of 82 studies measuring cytokine levels in healthy controls and participants with major depressive disorder (MDD), the latter had elevated peripheral levels of chemokine ligand 2, interleukin (IL)-1 receptor antagonist, 2, 6, 10, 12, 18, and tumor necrosis factor-alpha (TNF-α) and lower interferon-gamma (IFN-γ) levels (12). However, the direction of association between cytokine levels and severity of depression is ambiguous (9, 12, 15, 16). In patients with MDD, a linear correlation was observed between IL-1β, IL-8, and TNF-α and the severity of depression, whereas the transforming growth factor-beta (TGF-β) was significantly decreased in patients with chronic hepatitis B infection who had mild depression compared to those without depression and with moderate to severe depression (15, 16). Further evidence of the role of inflammation in psychiatric disorders has shown that anti-inflammatory agents influence changes in cytokine levels in MDD in a meta-analysis of clinical trials, indicating antidepressant effects (13, 17).

Neurobiological pathways involved in depression include inflammatory cytokine signals that initiate an inflammatory response in the brain and interfere with the activity of important behavioral regulatory neurotransmitters, including norepinephrine, dopamine, and serotonin (6, 18). Inflammatory cytokines can affect hypothalamic–pituitary–adrenal (HPA) regulation (18), causing the early onset of physical symptoms, such as pain, fatigue, loss of appetite, reduced exercise, sleep disorders, and reduced work performance and productivity (19). Prolonged activation of inflammation is detrimental to physical and mental well-being (19, 20). In recent years, nurses’ awareness of the importance of depression has increased because it can increase the risk of missed nursing care as well as threaten their own health (21–23).

The prevalence of depression is about twice as high among nurses as the general population (22, 24, 25). Nurses experience greater work stress than other healthcare professionals (21) and are at an increased risk of depression owing to shift work (26) and exhaustion owing to consistently caring for patients (27). Recently, researchers have identified a close association between depressive symptoms in nurses and abnormal eating habits (5, 28), increased risk of autoimmune diseases (29), and ovarian cancer (30). However, evidence for the role of inflammation in depression in the nurse population is limited. Therefore, understanding how depression is associated with inflammatory biomarkers among nurses and signaling the need for prevention and intervention are important.

The present study aimed to use a comprehensive immune-profiling approach to determine whether an abnormal profile of circulating cytokines could be identified in nurses with self-reported depression and whether this profile is associated with the severity of depression.

## 2. Methods

### 2.1. Study design and sample

The Korean Nurses’ Health Study is a prospective cohort study that began as a web-based survey of registered female nurses between the ages of 25 and 45 residing in Korea (31). It aimed to investigate the health status, lifestyle, health behavior, and illness of female nurses of childbearing age and identify industrial health, including work schedules, work conditions, work-related stress, and work risk exposure. A total of 20,613 registered female nurses responded to the basic questionnaire (Module 1) between July 2013 and November 2014. Participants in Module 1 were asked via text message to complete a follow-up online survey. Eight survey modules (Modules 2–9) were then opened to participants from 2014 to 2021.

In Module 5, 11,527 people participated in the survey from November 2016 to March 2017, and blood samples were collected from 1,703 nurses working in general hospitals who voluntarily agreed to provide blood. In this study, data from 1,703 individuals who provided blood samples in Module 5 and data from 157 individuals without missing data on key study variables, including cytokine levels, were used for analysis. Those who underwent medical diagnoses or suffered from mental disorders, pregnant or postpartum women, and those who had taken antidepressants or drugs with immune-regulatory effects such as glucocorticoids were excluded.

This study was approved by the Institutional Review Board of the affiliated university (IRB No. 117-4). Anonymity and confidentiality were assured, and informed consent was obtained from all participants.

### 2.2. Measurement of variables

In this study, the cytokine profile was used as the dependent variable, and depression was used as the independent variable. The covariates included personal characteristics, health behavioral factors, and work status.

#### 2.2.1. Depression

Depression was measured using the Patient Health Questionnaire (PHQ-9), a nine-item self-report measure that assesses the severity of depressive symptoms. It evaluates both physical and emotional depressive symptoms and corresponds to the DSM diagnostic criteria for MDD (21). It consists of a 4-point Likert scale ranging from 0 (not at all) to 3 (nearly every day) and has a score ranging from 0 to 27. Higher scores indicated greater symptoms of depression. The PHQ-9 comprises five categories of depression severity: none-minimal [0–4], mild [5–9], moderate [10–14], moderately severe [15–19], and severe [20–27] (32). In our study, Cronbach’s alpha for the PHQ-9 was 0.95.

#### 2.2.2. Cytokine profile analysis

Fasting peripheral blood samples were collected via intravenous puncture. After separating the serum through centrifugation, it was stored at −80°C until analysis. All samples were analyzed under the same conditions on the same day. Sixteen cytokine immunoassays were performed using the Luminex multiplexing system with the magnetic bead method and read on Luminex xMAP (Komabiotech, Seoul, Korea). Custom kits, including chemokine (C-C motif) ligand 2 (CCL2), chemokine (C-E-C motif) ligand 1 (CXCL1), interferon gamma (IFN-γ), IL-1RA, IL-1β, IL-2, IL-4, IL-5, IL-6, IL-7, IL-8, IL-10, IL-12, IL-18, transforming growth factor-alpha (TGF-α), and TNF-α, were used. The results are presented as the concentration of cytokines in the serum (pg/mL).

#### 2.2.3. Covariate information

To investigate the association between depression and cytokine profiles, covariates, including sociodemographic characteristics, health behavior, and work-related factors, were adjusted based on prior literature. Demographic factors included age (12, 26, 33), level of education (34), and marital status (34). Health behavioral factors included alcohol consumption (35) and body mass index (13, 26), and rotational shift work (26) was included as a work-related factor. None of the participants smoked; therefore, smoking was not included as a covariate.

### 2.3. Data analysis

Statistical analysis was performed using SPSS version 26.0 (IBM Corp., Armonk, NY, United States) based on a significance level of α = 0.05. Descriptive statistics were used for absolute and relative proportions for categorical data and means and standard deviations for continuous variables. One-way analyses of variance and Chi square test were used to investigate quantitative differences in the severity of depression. The two-sample Student’s *t*-test was performed to compare the serum levels of cytokines according to the severity of depressive symptoms (“no” vs. moderately severe, “no” vs. severe); variables were natural log or square root transformed in the final model if required to meet the normality assumption for the analyses.

To measure the effect of depression levels on cytokine profiles, we performed generalized gamma regression with the severity of depression = no as a reference variable. We controlled for factors related to demographic characteristics, health behavior, and rotational shift work. Gamma regression is a generalized linear model that is evaluated as an appropriate model when data are skewed to the right of non-negative and heteroscedasticity. Compared to regression analysis, which transforms the dependent variable using an exponential function, it does not require transformation and is easier to interpret (36, 37). Gamma regression was chosen because our cytokine data showed non-normal unequal variances (38, 39).

## 3. Results

### 3.1. Comparison of the demographic characteristics of participants

The mean age of the participants was 32 years (standard deviation, 5.9). The majority of 157 nurses were unmarried (59.9%) and had bachelor’s degrees (63.7%). Most participants had shift work (75.8%). The demographic characteristics of the depression-severity groups are shown in Table 1. There was no significant difference between the groups in age, marital status, level of education, alcohol consumption, body mass index, and rotational shift work (*p* > 0.05). Depression levels in the PHQ-9 are classified into five categories according to the score; however, our study includes only three categories, as none of our participants had either mild or moderate depression. The severity of depressive symptoms was as follows: 98 (62.4%) participants had none-minimal depression (2.03 ± 1.45), 44 (28.0%) had moderately severe depression (16.45 ± 1.34), and 15 (9.6%) had severe depression (22.13 ± 2.13).

**Table 1:** Demographic characteristics of participants.

Demographics	All (*n* = 157)	Severity of depression; *n* (%) of nurse			*F*	*p*
		None-minimal (*n* = 98)	Moderately-severe (*n* = 44)	Severe (*n* = 15)		
Age, years, mean ± SD	32.3 ± 5.9	32.7 ± 6.0	31.9 ± 6.3	31.3 ± 4.2	0.461	0.632
Marital status					1.489	0.229
Single	94 (59.9)	54 (55.1)	31 (70.5)	9 (60.0)		
Married	63 (40.1)	44 (44.9)	13 (29.5)	6 (40.0)		
Level of education					0.199	0.819
3-year college	34 (21.7)	22 (22.4)	8 (18.2)	4 (26.7)		
4-year college	100 (63.7)	63 (64.3)	29 (65.9)	8 (53.3)		
Master’s or higher	23 (14.6)	13 (13.3)	7 (15.9)	3 (20.0)		
Alcohol consumption					0.319	0.728
Never	33 (21.0)	21 (21.4)	8 (18.2)	4 (26.7)		
Occasionally	105 (66.9)	64 (65.3)	31 (70.5)	10 (66.7)		
Frequently	19 (12.1)	13 (13.3)	5 (11.4)	1 (6.7)		
Body mass index					0.936	0.394
Underweight	15 (9.6)	11 (11.2)	2 (4.5)	2 (13.3)		
Normal	90 (57.3)	52 (53.1)	30 (68.2)	9 (60.0)		
Overweight	51 (32.5)	35 (35.7)	12 (27.3)	4 (26.7)		
Shift work					0.647	0.525
No	38 (24.2)	26 (26.5)	10 (22.7)	2 (13.3)		
Yes	119 (75.8)	72 (73.5)	34 (77.3)	13 (86.7)		
PHQ-9 score, mean ± SD		2.03 ± 1.45	16.45 ± 1.34	22.13 ± 2.13	−38.705	<0.001^**^

** *p* < 0.01; PHQ-9, Patient Health Questionnaire; SD, standard deviation.

### 3.2. Comparison of cytokine profile by the severity of depressive symptoms

**Figure 1:**
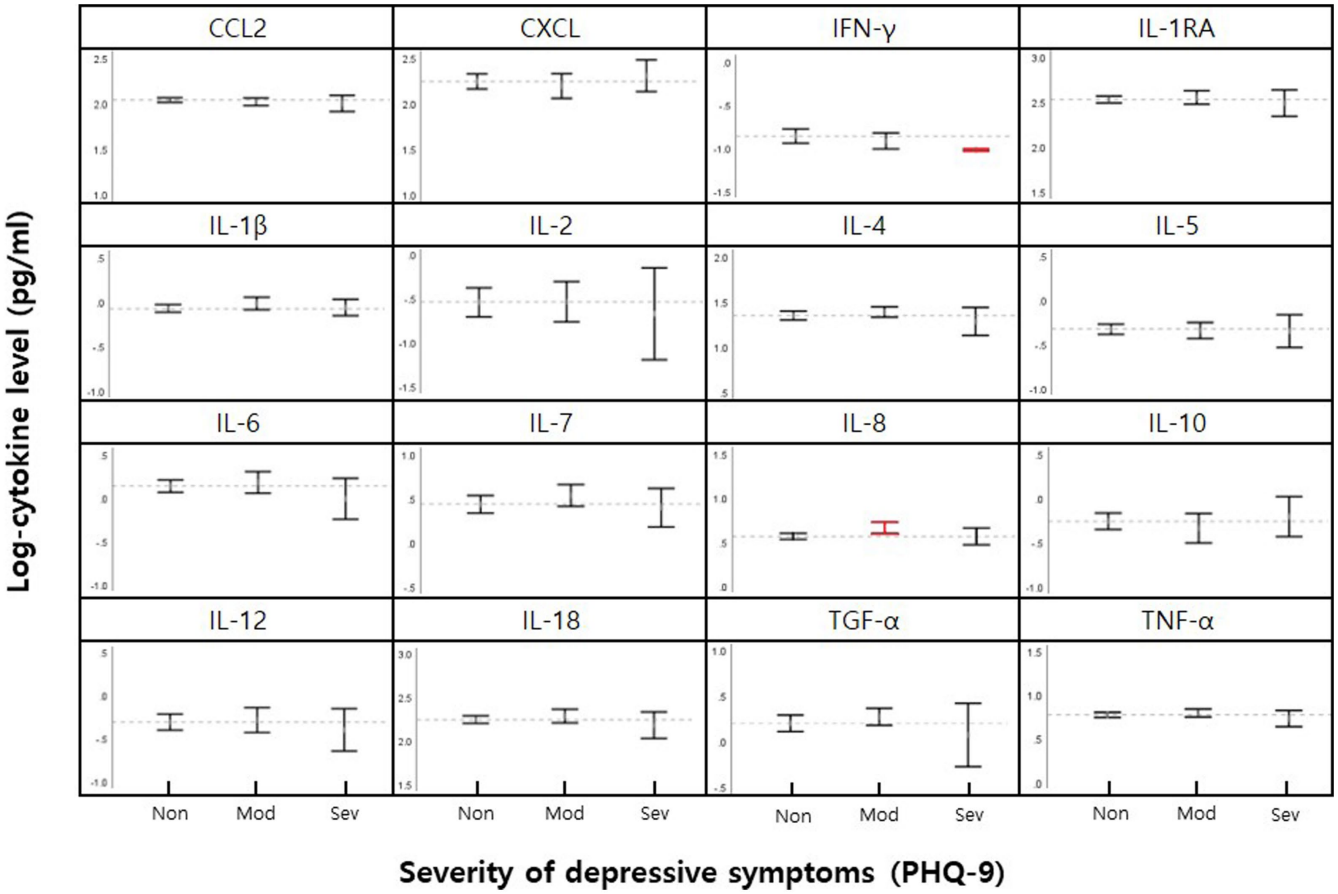
Mean cytokine levels in female nurse grouped by none-minimal (Non), moderately-severe (Mod), and severe (Sev) depressive symptoms. 95% CI for means for each cytokine are shown within vertical brackets. The dotted horizontal line within each cytokine panel represents the average value for no depressive symptoms. Statistically significant comparisons of depression severity level (*p* < 0.05, Table 2) are in red.

The serum levels of 16 cytokines according to the severity of depression are shown in Figure 1; Table 2. IL-8 levels were significantly different when stratified by severity, as shown in Figure 1 in red (*p* = 0.046).

**Table 2:** Comparison of mean cytokine levels in female nurses.

	Severity of depressive symptoms			Non vs. Mod	Non vs. Sev
	None-minimal	Moderately-severe	Severe		
Cytokine	Mean (95% CI)	Mean (95% CI)	Mean (95% CI)	*p*	*p*
CCL2	2.039 (2.013; 2.064)	2.020 (1.978; 2.061)	2.001 (1.912; 2.090)	0.418	0.300
CXCL1	2.243 (2.161; 2.326)	2.193 (2.058; 2.326)	2.305 (2.132; 2.478)	0.512	0.579
IFN-γ	−0.858 (−0.941; −0.776)	−0.914 (−1.007; −0.821)	−1.018 (−1.031; −1.006)	0.423	<0.001^**^
IL-1RA	2.529 (2.491; 2.567)	2.552 (2.478; 2.626)	2.489 (2.342; 2.636)	0.545	0.475
IL-1β	−0.072 (−0.115; −0.029).	−0.017 (−0.087; 0.054)	−0.062 (−0.154; −0.029)	0.168	0.865
IL-2	−0.544 (−0.708; −0.022)	−0.537 (−0.765; 0.308)	−0.673 (−1.194; 0.152)	0.963	0.577
IL-4	1.350 (1.302; 1.399)	1.389 (1.332; 1.446)	1.289 (1.130; 1.440)	0.346	0.344
IL-5	−0.327 (−0.385; −0.270)	−0.341 (−0.432; −0.251)	−0.348 (−0.533; −0.163)	0.793	0.798
IL-6	0.137 (0.066; 0.208)	0.180 (0.055; 0.305)	−0.008 (−0.243; 0.227)	0.526	0.154
IL-7	0.436 (0.336; 0.536)	0.537 (0.414; 0.660)	0.399 (0.181; 0.618)	0.238	0.787
IL-8	0.569 (0.534; 0.604)	0.665 (0.603; 0.727)	0.567 (0.473; 0.662)	0.004^*^	0.974
IL-10	−0.258 (−0.351; −0.165)	−0.337 (−0.503; −0.170)	−0.206 (−0.432; 0.021)	0.376	0.683
IL-12	−0.311 (−0.403; −0.218)	−0.287 (−0.430; −0.218)	−0.206 (−0.432; 0.021)	0.775	0.480
IL-18	2.243 (2.200; 2.286)	2.284 (2.205; 2.362)	2.179 (2.027; 2.331)	0.325	0.301
TGF-α	0.199 (0.109; 0.290)	0.271 (0.177; 0.364)	0.072 (−0.275; 0.419)	0.342	0.337
TNF-α	0.770 (0.740; 0.799)	0.791 (0.746; 0.836)	0.728 (0.635; 0.820)	0.422	0.312

^*^p < 0.05; ^**^p < 0.01; CCL, chemokine ligands; IFN-γ, interferon-gamma; IL, interleukins; TGF-α, transforming growth factor-alpha; TNF-α, tumor necrosis factor-alpha; SD, standard deviation; 95% CI, confidence interval; Non, none-minimal; Mod, moderately-severe; Sev, severe.

Grouped into none-minimal, moderately-severe, and severe depressive symptoms; *p*-value in the unadjusted analysis using two-sample students’ *t*-test of equal change.

### 3.3. Association between cytokine and depressive symptoms

Table 3 shows the results of analyzing the association between cytokine profiles and severity of depression using generalized gamma regression analyses. After controlling for all covariates, we found that the moderately severe depression group had increased IL-6 (*p* = 0.003), IL-8 (*p* = 0.001), and IL-18 (*p* = 0.023) levels but decreased IFN-γ (*p* = 0.003) levels. The severe depression group had lower levels of IFN-γ (*p* < 0.001) than the no-depressive symptom group.

**Table 3:** Association with cytokine level and the severity of depressive symptoms.

	Moderately-severe			Severe		
Cytokine	*B*	95% CI	*p*	*B*	95% CI	*p*
CCL2	−0.023	−0.129; 0.083	0.672	−0.063	−0.224; 0.099	0.446
CXCL1	−0.066	−0.369; 0.238	0.671	−0.063	−0.523; 0.397	0.788
IFN-γ	−0.585	−0.969; −0.201	0.003^**^	−1.254	−1.837; −0.672	<0.001^**^
IL-1RA	0.136	−0.050; 0.322	0.151	−0.011	−0.292; 0.271	0.942
IL-1β	0.135	−0.058; 0.329	0.171	−0.065	−0.358; 0.228	0.663
IL-2	−0.132	−0.570; 0.306	0.555	−0.092	−0.756; 0.572	0.786
IL-4	0.046	−0.119; 0.211	0.588	−0.137	−0.385; 0.112	0.280
IL-5	−0.070	−0.282; 0.141	0.514	−0.072	−0.393; 0.249	0.660
IL-6	0.460	0.159; 0.762	0.003^**^	−0.267	−0.724; 0.191	0.253
IL-7	0.114	−0.175; 0.402	0.441	−0.218	−0.656; 0.220	0.329
IL-8	0.273	0.118; 0.428	0.001^**^	−0.002	−0.237; 0.233	0.986
IL-10	−0.081	−0.426; 0.265	0.647	0.004	−0.519; 0.528	0.987
IL-12	−0.045	−0.460; 0.370	0.833	−0.211	−0.841; 0.418	0.510
IL-18	0.236	0.032; 0.440	0.023^*^	−0.137	−0.447; 0.172	0.385
TGF-α	0.142	−0.114; 0.397	0.277	−0.102	−0.489; 0.286	0.607
TNF-α	0.046	−0.071; 0.163	0.442	−0.085	−0.263; 0.092	0.347

^*^p < 0.05; ^**^p < 0.01; CCL, chemokine ligands; IFN-γ, interferon-gamma; IL, interleukins; TGF-α, transforming growth factor-alpha; TNF-α, tumor necrosis factor-alpha; 95% CI, confidence interval.

## 4. Discussion

This study investigated the association between depression severity and inflammatory cytokine profiles among female nurses of childbearing age. We found that self-reported depressive symptoms were associated with pro-inflammatory cytokines. Serum IL-6, IL-8, IL-18, and IFN-γ levels are estimated biomarkers for depression severity in nurses, showing that they may increase the risk of inflammatory dysregulation when nurses have high depressive symptoms. Recent changes in peripheral cytokines and chemokines in depression have shown that patients with MDD have increased average levels of pro-inflammatory immune markers and decreased levels of anti-inflammatory immune markers (12, 13). In our study, moderately severe depressive symptoms in nurses were associated with elevated levels of IL-6, IL-8, and IL-18, whereas IFN-γ showed the opposite profile. Participants with severe depressive symptoms showed decreased levels of IL-6, IL-8, IL-18, and IFN-γ. These results suggest a state of immune system dysregulation.

Increased levels of pro-inflammatory cytokines IL-6, IL-8, and IL-18 in nurses with moderately-severe depressive symptoms support a meta-analysis of inflammatory markers in depression studies that identify depression as a pro-inflammatory state (12). During acute infection, dendritic cells and macrophages produce IL-6, which is secreted in response to acute inflammatory stimulation (12). IL-8, which plays a pro-inflammatory role, mediates the movement of neutrophils to the inflammatory site as chemokines and influences the immune response in the acute inflammatory stage (40). IL-18 may have a significant effect on the pathophysiology of the CNS and contribute to neuro-inflammation (41). Higher levels of depression symptoms were associated with increased levels of pro-inflammatory biomarker IL-6 in caregivers caring for an older person in the community (42). Pregnant women with severe anxiety and accompanying depressive symptoms showed a significant increase in serum levels of IL-6 and TNF-α (43). Similar to our results, this suggests that nurses with high depressive symptoms are at risk of increased inflammation, and that changes in cytokine concentrations may be affected by the intensity of depressive symptoms.

Contrastingly, the blood samples of nurses with severe depression in our study showed a decrease in pro-inflammatory cytokine levels. Regarding the severity of depression, some studies did not find a relationship between cytokines and disease severity (44, 45) or showed a negative correlation between serum cytokine levels and depression severity in patients with major depression (46), pregnant women with depressive symptoms (47), and patients with breast cancer (48). In the present study, IL-6, IL-8, and IL-18 levels increased with moderately severe depressive symptoms but decreased with severe depressive symptoms. This finding suggests the dysregulation of the HPA axis. The hypothalamus is the central site for regulating autonomous body functions and adapting behavior to environmental stimuli and is involved in depression pathology (49). The interaction between cytokines and HPA activity has been observed to be dependent on depression (50). Adaptation of the HPA response was maximized when faced with a severe stressor, and the HPA response to a stressor repeated daily was shown to decrease gradually (51). In other words, a state of reduced immune response is considered to result from a blunt HPA axis response. However, the validation of these results requires further research with broader sample profiles.

In our study, the level of IFN-γ decreased in both depressive and non-depressive symptom groups. This was consistent with the meta-analysis results of 17 studies showing that patients with MDD had decreased IFN-γ levels compared with healthy controls (13). However, another meta-analysis that considered smoking status reported increased IFN-γ levels (12). The nurses in the study were in a controlled, tobacco-free state, and the results of IFN-γ were more controversial. Given that it has not previously been consistent in a smaller meta-analysis (52) related to IFN-γ, we believe that more studies on IFN-γ in relation to depression are needed.

Both TNF-α and IL-2 are well known pro-inflammatory cytokines that play a central role in the early stages of the immune response, highlighting the systemic nature of inflammatory conditions (53). Higher levels of depression symptoms were associated with increased levels of pro-inflammatory biomarkers CRP and TNF-α in older nurses working in the United States (54). In this study, no significant results were observed for TNF-α and IL-2 levels; however, there exists an inverted U-shape relationship between the severity of depressive symptoms and TNF-α and IL-2 levels. These results may support the notion that chronic stress does not control immune function but may lead to the suppression of the immune response (53). However, further studies using longitudinal samples that can reflect acute and chronic stress are needed to confirm these findings.

Notably, the participants in this study were nurses with self-reported depressive symptoms, not diagnosed with depression. Because of their healthcare knowledge, nurses are more likely to engage in healthy lifestyle habits such as no smoking and regular exercise; however, because of work shifts, most of the time, they are involved in physically demanding tasks, such as standing for long. The nature of labor-intensive work can affect systemic inflammation and depression. Screening nurses for depression and providing early intervention may be ways to improve health before symptoms worsen, leading to physiological dysregulation.

This result should be considered in light of several limitations. First, although the Korean Nurses’ Health Study is a large cohort study, participants who provided blood were conveniently extracted and the sample size was relatively small. Additionally, the samples were all female, with a limited ability to investigate potential gender differences. Previous studies have shown sex differences in both depression and inflammation, and women are more likely to experience a more detrimental effect of depression on inflammation. Second, causality could not be inferred by considering the cross-sectional characteristics of the current study. Future longitudinal studies are needed to address whether nurses who self-reported depressive symptoms are within, or fluctuate between, cytokine signatures and disease severity categories over time. Finally, various cytokines have been studied; however, the results do not reflect changes in the overall immune network response, such as immune cell abnormalities. Further studies of other immune cells, such as the ratio of T-helper type-1 (Th1) cells to type-2 (Th2) cells are needed, which can provide a comprehensive understanding of changes in overall immune network responses.

## 5. Conclusion

Our study showed inflammatory cytokine profiles in female nurses with depressive symptoms. Serum IL-6, IL-8, IL-18, and IFN-γ are estimated biomarkers for depression and can identify the physiopathology of inflammatory regulation abnormalities in depression. Current research also suggests that more attention should be paid to depression among nurses, given that most nurses do not seek mental health services for depression. We propose early detection and intervention, considering the mechanisms linking depression to physical illness and disease.

## Data availability statement

The original contributions presented in the study are included in the article/supplementary material, further inquiries can be directed to the corresponding author.

## Ethics statement

The studies involving human participants were reviewed and approved by the institutional review board of the Ewha Womans University (No. 117-4). The patients/participants provided their written informed consent to participate in this study.

## Author contributions

YK, YP, OK, and HL designed the study, collected the data, and interpreted the results. YK and HL interpreted data, wrote the manuscript, interpreted the results, and discussed and revised the manuscript. HP collected and organized the biological samples and performed and interpreted the cytokine assay. YP and OK organized the survey, collected data, and discussed and revised the manuscript. All authors contributed to the article and approved the submitted version.

## Funding

The Korean Nurses’ Health Study received financial support to conduct research from the Korea Disease Control and Prevention Agency of the Korea National Institute of Health. This research was supported by a grant from the Korea Disease Control and Prevention Agency (2016ER630500, 2016ER630501 and 2022ER0602-01).

## Conflict of Interest

The authors declare that the research was conducted in the absence of any commercial or financial relationships that could be construed as a potential conflict of interest.

## Publisher’s note

All claims expressed in this article are solely those of the authors and do not necessarily represent those of their affiliated organizations, or those of the publisher, the editors and the reviewers. Any product that may be evaluated in this article, or claim that may be made by its manufacturer, is not guaranteed or endorsed by the publisher.

## Abbreviations

CCL: Chemokine ligands
HPA: Hypothalamic-pituitary-adrenal
IFN-γ: Interferon-gamma
IL: Interleukins
MDD: Major depressive disorder
PHQ-9: Patient Health Questionnaire
TGF-α: Transforming growth factor-alpha
TGF-β: Transforming growth factor-beta
Th1: T-helper type-1
Th2: T-helper type-2
TNF-α: Tumor necrosis factor-alpha
